# On the Single-Crystal Structure of Tenofovir Alafenamide Mono-Fumarate: A Metastable Phase Featuring a Mixture of Co-Crystal and Salt

**DOI:** 10.3390/ijms21239213

**Published:** 2020-12-03

**Authors:** Jian-Wei Wang, Lu Liu, Ka-Xi Yu, Hong-Zhen Bai, Jun Zhou, Wen-Hua Zhang, Xiurong Hu, Guping Tang

**Affiliations:** 1 Department of Chemistry, Zhejiang University, Hangzhou 310028, China; wjw126hz@zju.edu.cn (J.-W.W.); lulu0698@126.com (L.L.); yukaxi@zju.edu.cn (K.-X.Y.); hongzhen_bai@zju.edu.cn (H.-Z.B.); zerobomp@zju.edu.cn (J.Z.); huxiurong@zju.edu.cn (X.H.); 2 College of Chemistry, Chemical Engineering and Materials Science, Soochow University, Suzhou 215123, China; whzhang@suda.edu.cn

**Keywords:** tenofovir alafenamide, pharmaceutical co-crystal, fumaric acid, anti-HIV drug, chronic hepatitis B, Hirshfeld analysis, hydrogen-bonding, nucleotide reverse transcriptase inhibitor

## Abstract

Tenofovir alafenamide (TAF) is a prodrug of tenofovir as a potent nucleotide reverse transcriptase inhibitor. It serves as the key component of Genvoya^®^ for the first-line treatment of human immunodeficiency virus infection (HIV) and is the active component of Vemlidy^®^ for the treatment of chronic hepatitis B. Vemlidy^®^ is also a monotherapeutic regimen formulated as TAF hemifumarate (**1**; TAF:fumarate = 2:1). In this work, we report for the first time the single-crystal structure of TAF fumarate hemihydrate (**2**, TAF:fumarate:H_2_O = 2:2:1). Compound **2** is initially documented as a salt in which one proton of the fumaric acid migrates to the amine group of the adenine moiety in TAF. It was recently proposed that ca. 20–30% proton is transferred to the N atom on the aromatic adenine backbone. We herein provide definitive single-crystal X-ray diffraction results to confirm that **2**, though phase pure, is formed as a mixture of co-crystal (75%) and salt (25%). It features two pairs of TAF fumarates, wherein one of the four H atoms on the fumaric acid is transferred to the N atom of the adjacent adenine moiety while the other three carboxylates remain in their intrinsic acid form. Compound **2** is a metastable phase during the preparation of **1** and can be isolated by halting the reaction during the refluxing of TAF and fumaric acid in acetonitrile (MeCN). Our report complements the previous characterizations of TAF monofumarate, and its elusive structural patterns are finally deciphered.

## 1. Introduction

Human immunodeficiency virus infection (HIV), also known as acquired immune deficiency syndrome (AIDS), is an infectious disease that involves the production of new DNA genome from RNA genome via the virus’s own reverse transcriptase enzyme [1,2,3,4,5,6]. During the HIV infecting process, the virus can integrate its DNA (produced via a retroviral process) to the host cell genome by an integrase enzyme, which leads to difficulty in curing HIV [7]. HIV is thus globally one of the major causes of death, featuring a mortality of 770,000 among 38 million infected in 2018 [8].

**Figure 1:**
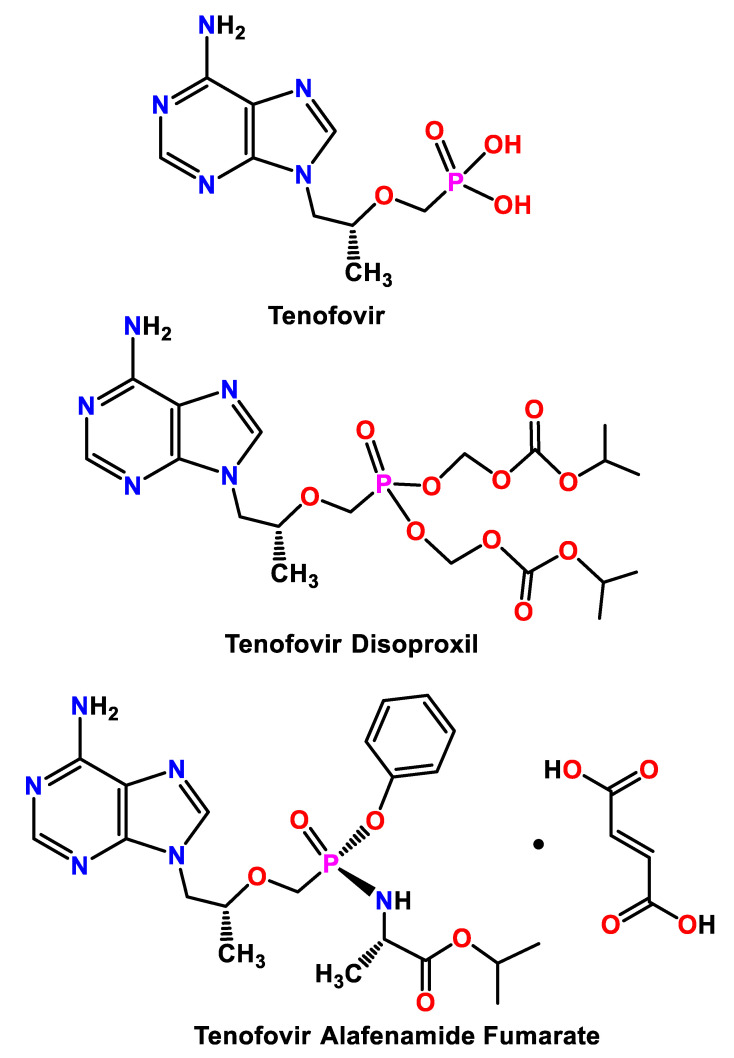
The structures of tenofovir, tenofovir disoproxil, and tenofovir alafenamide fumarate

In current medical practice, inhibiting the retroviral process is a major approach to suppressing the replication of viruses to minimize liver damage when modulating immune system dysfunction in HIV [9]. In this regard, tenofovir (TF, Figure 1) is a nucleotide reverse transcriptase inhibitor, which is metabolized intracellularly by the lysosomal protease cathepsin A, adenylate kinases, and nucleotide kinases to form the active moiety tenofovir diphosphate [4,10,11,12].

Accordingly, TF derivatives, particularly tenofovir disoproxil (TDF, Figure 1), in combination with the other drugs cobicistat, elvitegravir, emtricitabine, and rilpivirine, have been extensively exploited as prodrugs of TF for the treatment of HIV-1 infections [11,13]. Compared to the native TF, TDF is designed to improve TF permeability, allowing a systemic drug delivery via oral administration. However, TDF is quickly metabolized to TF by gut and serum esterases in vivo, and a high drug exposure is thus needed to allow the proper loading of target cells, raising concerns about side effects such as renal and bone toxicity [12].

Recently, tenofovir alafenamide (TAF, Figure 1) has been developed as an important surrogate for TDF due to its enhanced stability in blood and plasma, which leads to a longer circulation time [4,14]. Besides, TAF is readily transformed into TF within lymphocytes, providing improved intracellular drug accumulation in HIV target cells. Furthermore, TAF is more stable in blood when compared to TDF and efficiently delivers tenofovir to cells with lower dosages, and it possesses a greater antiviral activity and better distribution into lymphoid tissues with a lower incidence of adverse side effects such as impaired kidney function and bone mineral density loss [11,14].

In the pharmaceutical industry, TAF salts (hydrochloride, oxalate, sulfate, and fumarate) were prepared in order to enhance the physicochemical properties of TAF, such as the stability, tableting and compression behavior, solubility, and dissolution profile [15,16,17]. In 2015, the Food and Drug Administration (FDA) approved TAF in combination with elvitegravir, cobicistat, and emtricitabine for the treatment of HIV-1 infection under the name of Genvoya^®^ [18,19]. Subsequently, in 2016, the FDA also approved TAF hemifumarate as the monotherapeutic regimen for the treatment of chronic hepatitis B under the name Vemlidy^®^ [18,20].

In a very recent report, TAF and TAF hemifumarate (**1**; TAF:fumarate = 2:1) have been prepared and structurally authenticated by single-crystal X-ray diffraction studies, with 1 also being confirmed as TAF and fumaric acid co-crystal [13]. However, when TAF was precipitated as monofumarate (TAF:fumarate = 1:1), three polymorphic forms existed, as revealed by powder X-ray diffraction (PXRD) [13]. The ^15^N solid-state nuclear magnetic resonance (NMR) of one of these forms also indicated that it contained ca. 20–30% salt, where the protons were on the aromatic N atoms of the adenine moiety while the other two phases featured exclusively as co-crystals. The monofumarate form of TAF was also originally documented as a salt, where one of the two fumaric acid protons migrated to the –NH_2_ group of the adenine backbone to give [–NH_3_]^+^ [21]. The single-crystal structure of **2**, and thus the correct associations between TAF and fumarate, are so far elusive.

Given the great promise of TAF in the treatment of HIV and chronic hepatitis B infections and the vital role of co-crystallization reagent (e.g., fumaric acid) in tuning the pharmacokinetics of active pharmaceutical ingredients (API), we managed to recrystallize TAF fumarate in nonconventional solvent dimethyl sulfoxide (DMSO) and obtain high-quality single-crystals of TAF fumarate in its hemihydrate form (**2**; TAF:fumarate:H_2_O = 2:2:1). The single-crystal X-ray diffraction analysis of **2** indicated that it featured a mixture of co-crystal (75%) and salt (25%). We herein elaborate on its single-crystal structure features and its supramolecular characteristics using a Hirshfeld surfaces analysis.

## 2. Results and Discussion

### 2.1. Synthesis and Spectroscopic Characterizations

**Figure 2:**
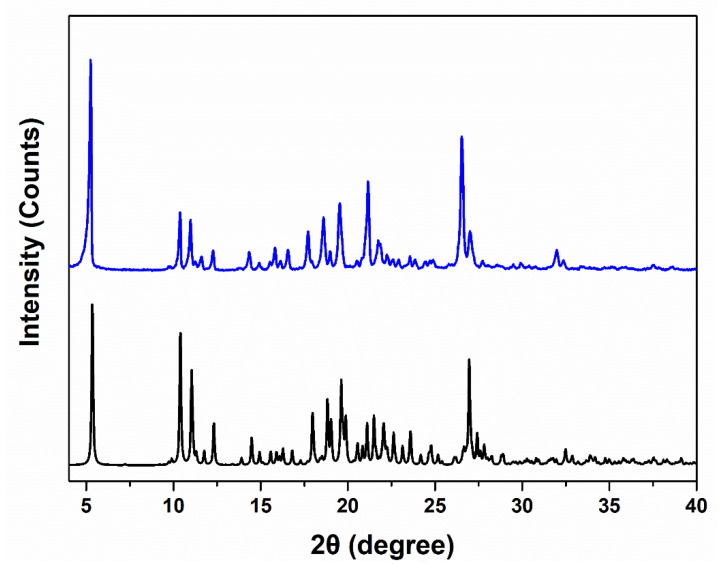
The powder X-ray diffraction (PXRD) patterns of **2** showing a good consistency between the experimental one (blue) and that simulated (black) from single-crystal diffraction data, and thus a high phase purity for **2**. Where 2Ɵ is the angle between transmitted X-ray beam and reflected beam.

By using nonconventional DMSO as the crystalline solvent, we successfully obtained single-crystals of TAF fumarate hemihydrate (**2**) as colorless block single crystals. As shown in Figure 2, the powder X-ray diffraction (PXRD) pattern of the bulk samples of **2** is in good agreement with that simulated from the single-crystal data, indicating that the formed crystals exhibit a high phase purity. The ^1^H NMR spectroscopy (Supplementary Figure S1) also indicates a 1:1 ratio for TAF and fumarate in **2**. It is notable that for the acid-base pair like **2**, proton transfer from fumaric acid to TAF may take place depending on their relative p*K*_a_ values [22]. When the p*K*_a_ (base)–p*K*_a_ (acid), denoted as ∆p*K*_a_, is greater 3, the formation of salt can be expected. On the contrary, when ∆p*K*_a_ is below 0, the proton transfer is inhibited and the co-crystals exist exclusively. The product outcome with ∆p*K*_a_ values between 0–3 remains hard to predict. Our present case is further complicated by two p*K*_a_ values (p*K*_a1_ = 3.0, p*K*_a2_ = 4.4) for fumaric acid and two p*K*_a_ values (p*K*_a1_ = 4.2, p*K*_a2_ = 9.8) for TAF [13]. We therefore invoked Fourier-transform infrared (FT-IR) spectra in combination with single-crystal X-ray diffraction results to study the structural features of **2**.

**Figure 3:**
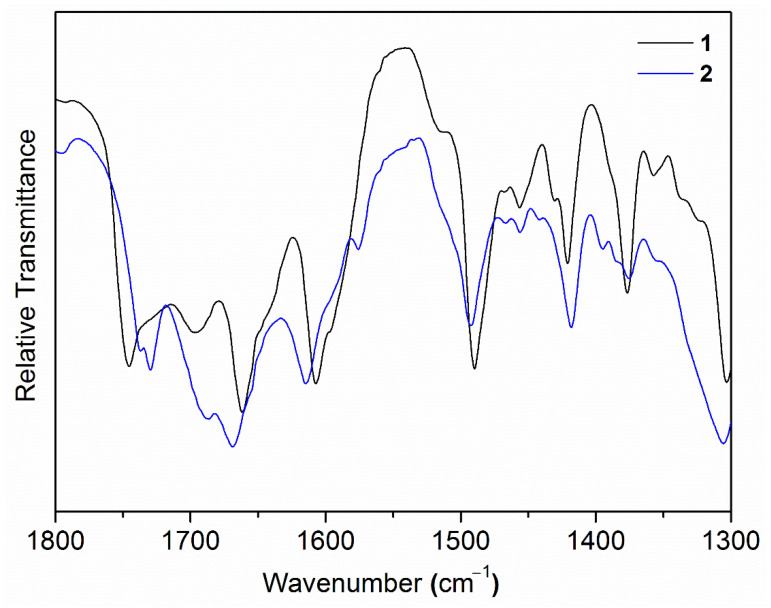
The Fourier-transform infrared (FT-IR) spectra of **1** (black) and **2** (blue) showing the relative intensity of the asymmetric (1607/1615 cm^−1^) and symmetric (1421/1418 cm^−1^) vibrations. The vibration intensity values at 1607 cm^−1^ and 1615 cm^−1^ were normalized for comparison purposes.

Comparatively, in the Fourier-transform infrared (FT-IR) spectra (Figure 3 and Supplementary Figure S2), peaks at 1607/1421 cm^−1^ (**1**) and 1615/1418 cm^−1^ (**2**) were assignable as the asymmetric/symmetric vibrations of COO^−^ in fumarate [23,24,25]. Notably, the symmetric vibration intensity in **2** is relatively stronger when compared to that of **1** (Figure 3), presumably indicating that **2** is rich in deprotonated and conjugated COO^−^.

The thermogravimetric analysis coupled with differential scanning calorimetry (TGA-DSC) reveals that compound **2** exhibits a smooth and continuous solvate loss before 120 °C (Supplementary Figure S3). This is followed by a plateau until 187 °C, at which point the rapid decomposition of **2** commences. Compound **2** loses 1.39% of its total mass before 120 °C, corresponding to the extrusion of the crystalline aqua solvate (calculated as 1.49%). For comparison, due to its solvate-free structure features, **1** shows almost no weight loss until decomposition starts at 186 °C.

### 2.2. Single-Crystal Structure Analysis of ***2***

**Figure 4:**
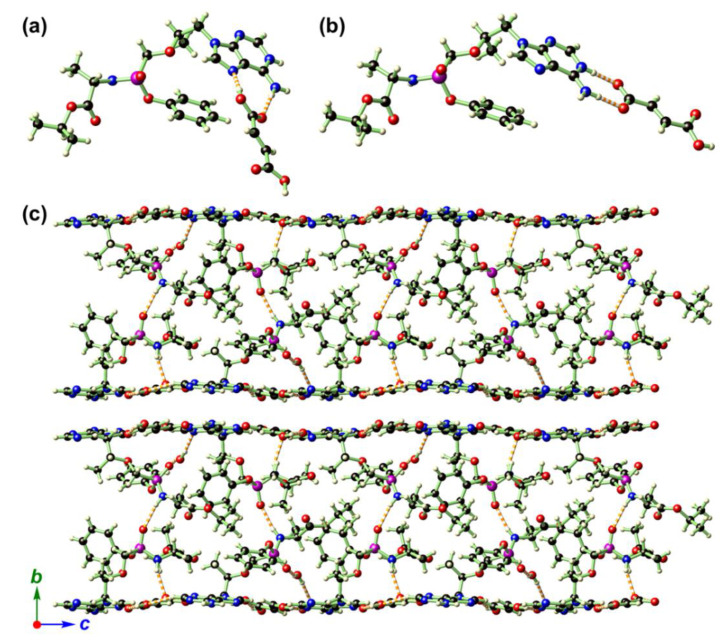
The structure of **2** showing (**a**,**b**) two independent TAF fumarate pairs in the asymmetric unit. In (**b**), the proton on the fumarate was found to migrate to the adenine moiety, and, as a result, the hydrogen-bonding modes in the two TAF fumarate pairs were drastically different. (**c**) depicts the crystal packing diagram of **2** when looking along the crystallographic *a* direction, showing its extensive hydrogen-bonding interactions and sandwich-type structure. Color codes: P (magenta), O (red), N (blue), C (black), H (light-yellow).

TAF (Figure 1) is a chemical entity that connects [(S)-1-(isopropoxycarbonyl)ethyl]amino-phenoxy and [(1R)-2-(6-amino-9H-purin-9-yl)-1-methylethoxy]methyl groups via a phosphinyl linkage. The ether and phosphinyl linkages provide the conformational flexibility of TAF, which allows the whole chain to adopt appropriate spatial orientations. Compound **2** crystallizes in the monoclinic space group *P*2_1_ with two TAF (Table 1), two fumarates, and one aqua molecule in its asymmetric unit (Figure 4a,b). To examine the protonation state of the carboxylates of the two fumarate moieties, we locate the protons from the difference Fourier map during the single-crystal X-ray crystallography and then allow the coordinates of these protons to be freely refined. It turned out that three protons were attached to the O atoms (O8A, O6B, and O8B) with O–H distances in the acceptable range of 0.83(5)–1.02(5) Å, while the fourth proton originally assigned to O6A migrated to the adjacent adenine moiety (N4B) with an ideal O–H distance of 0.83(4) Å. Thus within the two fumarates, three of the four carboxylates remain in acid form with an admixture of C–O and C=O bonds, and one remains deprotonated and in conjugated form.

Complementary to the proton locations, the respective C–O bond distances in these four carboxylates also vary considerably. For the three carboxylates in acid form, the C–O bonds can be categorized into two groups with a narrow bond range; the shorter distances in the range of 1.215(5)–1.227(5) Å are assignable as C=O double bonds, while the significantly longer distances in the range of 1.308(4)–1.314(4) Å are assignable as C–O single bonds (Supplementary Table S1). In sharp contrast, in the deprotonated and conjugated form of the carboxylate, the two C–O distances are 1.239(4) Å and 1.277(4) Å, which are close values. The large discrepancy in the bond distances within the carboxylate of the acid as well as the similar distances in the carboxylate in deprotonated form are similar to zwitterionic and hemizwitterionic coordination complexes such as *trans*-[Zn(*iso*-hmnH)_2_(H_2_O)_2_], *cis*-[Zn(hmnH)_2_(H_2_O)_2_]·2H_2_O, [CuCl(*iso*-hmnH_2_)(*iso*-hmnH)]·H_2_O, and [CuCl(hmnH_2_)(hmnH)] (*iso*-hmnH = 2-(hydroxymethyl) isonicotinate; hmnH = 2-(hydroxymethyl)nicotinate) [26,27,28]. The structure refinement results for the possible location of the protons, combined with the similarity and differences in the in C–O bond distances in the carboxylate, provide convincing evidence that the fumarate is present in both its acid (75%) and deprotonated (25%) forms, endorsing **2** as a mixture of both co-crystal and salt. These results support the previous report by Lengauer et al. that 20–30% proton was transferred to the N atom on the aromatic adenine backbone [13].

The orientation of the phenyl ring with respect to the adenine ring in **2** is defined by the dihedral angles between its least-square planes and features ca. 63.2° and 21.7° for the two TAF moieties in **2**. While one pair is similar to those found in TAF hemifumarate **1** (45.4°/79.9° for two disordered phenyl rings) and the pristine TAF (58.8°), the other pair deviates from these values significantly. These differences may result from the conformational flexibility provided by the ether linkages and the phosphinyl linkage, which can be quantified by the bond angles around the central P atom and the corresponding torsion angles along the six-membered chains (–O–P–C–O–C–C–; Supplementary Tables S1 and S2).

**Figure 5:**
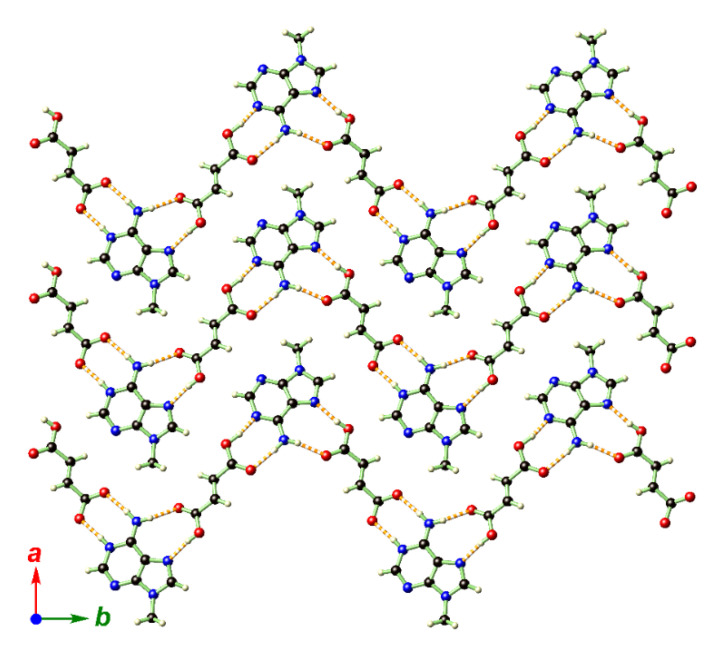
The hydrogen-bonded network layer in **2** extended within the *ab* plane and exclusively supported by the adenine moiety in TAF and fumarate. Color codes: O (red), N (blue), C (black), H (light-yellow).

The crystal packing in **2** is determined by N–H···O and O–H···N hydrogen bonds among TAF, fumarate, and H_2_O, giving a complicated two-dimensional (2D) network that is stacked along the crystallographic *b* direction (Figure 4c, Supplementary Table S3). It is interesting to note that the 2D hydrogen-bonded network also features a sandwich-like structure wherein all the flexible moieties around the central P atom (except the aromatic adenine ring) and aqua solvate are hosted by the hydrogen-bonded planes supported by adenine and fumarate (Figure 5).

**Figure 6:**
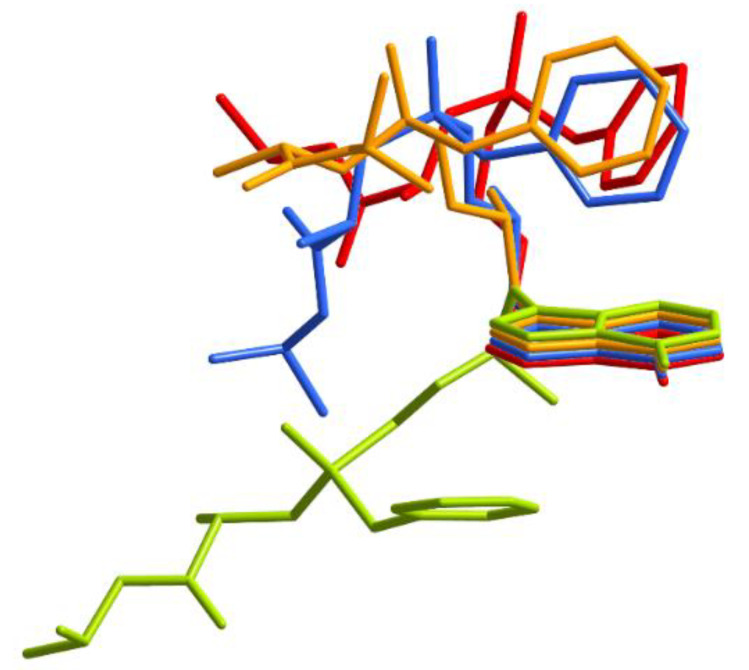
A superposition of the molecular conformations of the TAF moieties in their pristine form (red), **1** (light blue), and **2** (orange and lime), showing that one TAF in **2** exhibits drastically different conformations when compared to all the other forms.

In **2**, the two TAF molecules in its asymmetric unit exhibit an opposite configuration, with the P1–C13–O5–C14 torsion angles being −122.9 (3)° (for pristine TAF), −160.2 (5)° (1), and −149.9 (4)°/174.2 (4)° (2) (Supplementary Tables S1 and S2). To further elaborate the conformational variation of the TAF moieties in **2**, we overlayed the TAF moieties in the pristine TAF with those in **1** and **2** for comparison. As depicted in Figure 6, the conformational flexibility provided by the phosphinyl and ether linkages in the TAF molecule allows the phenyl ring, adenyl ring, and (S)-1-(isopropoxycarbonyl)ethyl]amino motif to adopt different orientations. One of the TAF conformations in **2** is rather different from all the others. 

### 2.3. Hirshfeld Surface Analysis of TAF, ***1***, and ***2***

**Figure 7:**
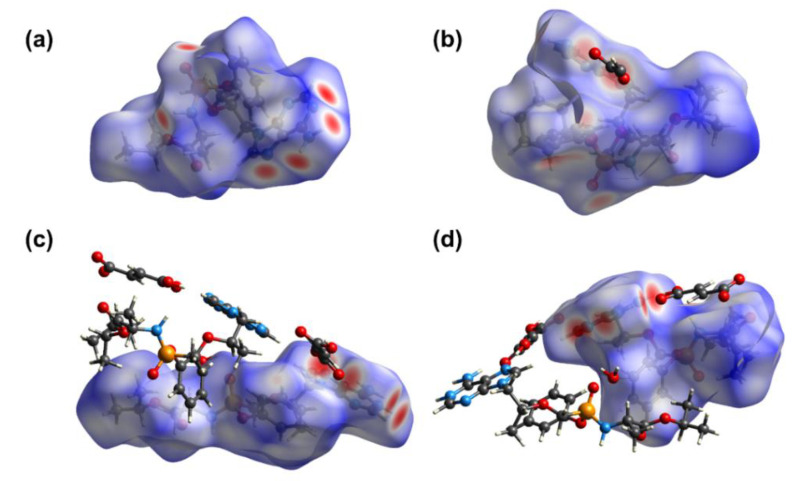
Hirshfeld surfaces for the TAF with its neighboring molecules in the crystals of (**a**) pristine TAF, (**b**) **1**, and (**c**,**d**) **2**, showing that two fumarate molecules in **2** were attached to one adenine ring at two different sites via hydrogen bonding.

Hirshfeld surface (HS) analysis is regarded as a useful tool for assessing the packing modes and intermolecular interactions in molecular crystals [29]. To evaluate and compare the nature of close interactions between the different lattice components that dictate the supramolecular architectures in TAF, **1,** and **2**, three-dimensional (3D) Hirshfeld surfaces and two-dimensional (2D) fingerprint maps were generated using the program Crystal Explorer [30]. The Hirshfeld surfaces of TAF and its fumarate are shown in Figure 7. The deep-red spots on the surface reveal the shortest N–H∙∙∙N, N–H∙∙∙O, O–H∙∙∙N, and O–H∙∙∙O interactions. Other visible spots on the surfaces correspond to subtle H∙∙∙H contacts. Overall, the HS of TAF, **1**, and **2** differ from each other in shape, reflecting the different weights of intermolecular contacts.

**Figure 8:**
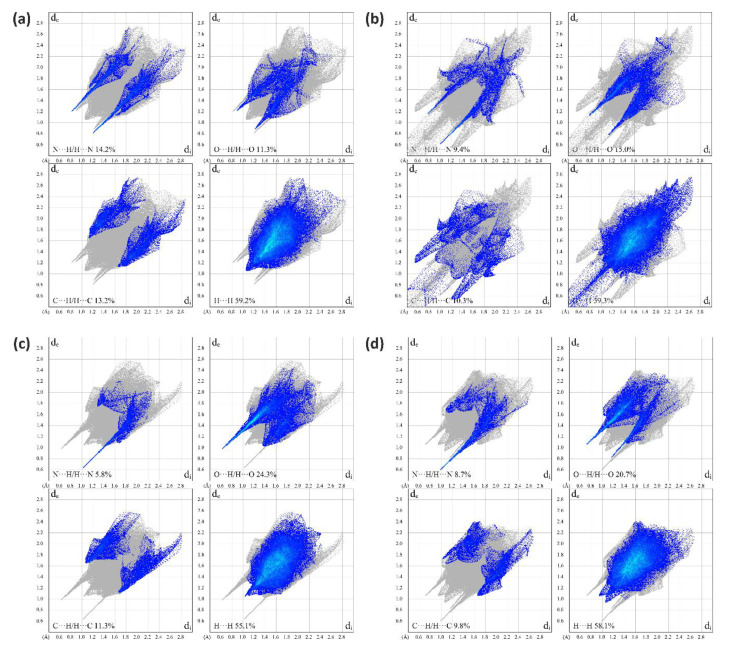
Two-dimensional fingerprint plots of (**a**) TAF, (**b**) **1**, and (**c**,**d**) **2**, showing that the plots of the two spatially unique molecules in **2** possess a high similarity when compared to that of pristine TAF and **1**.

**Figure 9:**
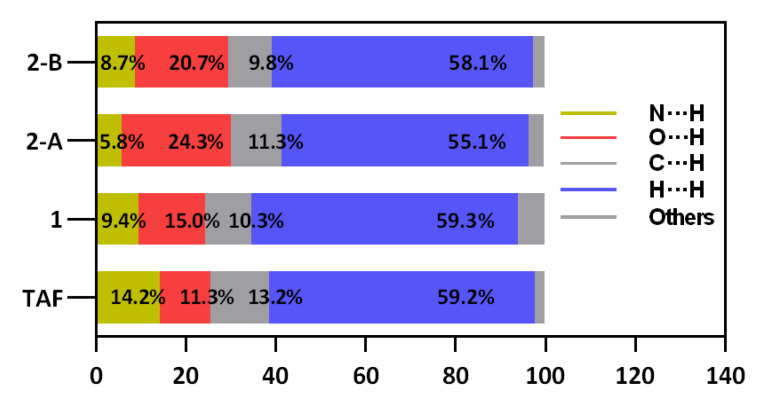
Relative ratios of the contributions of different interactions to the Hirshfeld surface for TAF, **1**, and two TAF-fumarate pairs in **2** (denoted as **2**-A and **2**-B), showing that the presence of water molecules in **2** greatly raises the relative contribution of O∙∙∙H/H∙∙∙O amongst the collection of interactions.

A summary of intermolecular contacts for TAF and its fumarates are shown with decomposed fingerprint plots (Figure 8) to emphasize detailed atom-pair close contacts, including H∙∙∙H, O∙∙∙H/H∙∙∙O, C∙∙∙H/H∙∙∙C, and N∙∙∙H/H∙∙∙N. Intermolecular interactions appear as a discrete spike in the 2D fingerprint plots. The quantitative analysis (summarized in Figure 9) shows that the H∙∙∙H interactions are the largest contributor to the total HS for TAF, **1**, and **2** relative to other contacts, showing large surfaces in the middle of scattered points in the fingerprint plots. As a result of the existence of a fumaric acid/fumarate anion in **1** and **2** (also with one additional aqua molecule), the N∙∙∙H/H∙∙∙N interactions in **1** and **2** are a smaller contributor to the total HS than those in TAF. In compliance with the single-crystal structure analysis, the O∙∙∙H/H∙∙∙O interactions have a secondary contribution in **1** and **2**, followed by the C∙∙∙H/H∙∙∙C interactions.

## 3. Materials and Methods

### 3.1. General

Tenofovir alafenamide hemifumarate (**1**) was kindly donated by Hangzhou Zhongmeihuadong Pharmaceutical Co.,Ltd (Zhejiang, China) and used without further purification. All the other chemicals were obtained directly from commercial sources and used as received. The ^1^H nuclear magnetic resonance (NMR) spectra were recorded on a Bruker DRX-500 spectrometer (Ettlingen, Germany), and chemical shifts (δ) are referenced to the residual solvent peaks or internal standard TMS. FT-IR spectra were measured on a Thermo Scientific Nicolet iS50 FT-IR spectrometer (Waltham, MA, USA) as KBr disks (400–4000 cm^−1^). Powder X-ray diffraction (PXRD) patterns were recorded on a Rigaku D/Max-2550PC microdiffractometer (Tokyo, Japan). A rotating-anode Cu-target X-ray (λ = 1.5406 Å) was used, which was worked at 40 kV, 250 mA with the scanning ranges of 3.0 to 40.0° and a scanning speed of 5° min^−1^ with an increasing step size of 0.02° and count time of 0.5–2 s. The TGA-DSC investigation was performed on a TA DSC Q100 differential filtering calorimeter (New Castle, PA, USA) at a heating rate of 10 °C min^−1^ under a nitrogen stream of 50 cm^3^ min^−1^.

### 3.2. Crystallization of TAF Fumarate and Its Characterization Data

The bulk powdery sample of **2** was prepared by heating **1** (0.647 g, 1.21 mmol) and fumaric acid (0.173 g, 1.49 mmol) in acetonitrile (MeCN) (15.7 mL) at 82 °C for 8 min, followed by immediate filtration and cooling at r.t. Single crystals were obtained by dissolving the as-synthesized powdery sample of **2** in DMSO at 82 °C and then allowing the over-saturated solution to stand at r.t. for one week to yield the plate-like single crystals. ^1^H NMR (500 MHz, DMSO-*d*_6_): δ 13.14 (s, 2H), 8.14 (s, 1H), 8.11 (s, 1H), 7.31–7.28 (m, 2H), 7.22 (s, 2H), 7.15–7.12 (m, 1H), 7.06–7.04 (m, 2H), 6.63 (s, 2H), 5.63 (m, 1H), 4.85 (sep, J = 6.25 Hz, 1H), 4.29–4.26 (dd, 1H), 4.17–4.13 (dd, 1H), 3.97–3.91 (m, 1H), 3.90–3.81 (m, 2H), 3.79–3.74 (dd, 1H), 1.16–1.15 (d, J = 6.25 Hz, 6H), 1.14–1.12 (d, J = 7.2 Hz, 3H), 1.08–1.06 (d, J = 6.2 Hz, 3H). IR (KBr disk): 3327 (s), 3176 (s), 2979 (m), 2983 (m), 2517 (m), 1930 (w), 1795 (w), 1736 (s), 1730 (s), 1687 (s), 1669 (s), 1615 (s), 1576 (w), 1492 (m), 1456 (w), 1418 (m), 1375 (m), 1306 (s), 1232 (m), 1208 (s), 1183 (s), 1149 (s), 1105(s), 1067 (m), 1021 (m), 984 (m), 918 (s), 784 (w), 759 (m), 721 (w), 690 (m), 647 (m), 597 (w), 579 (w), 560 (m), 499 (w), 477 (w) cm^−1^.

### 3.3. X-ray Crystallography

The single crystal of **2** was analyzed on a Rigaku RAXIS-RAPID CCD X-ray diffractometer (Rigaku Corporation, Tokyo, Japan) at 296(2) K with graphite monochromated Mo Kα (λ = 0.71073 Å) radiation with absorption correction (multiscan) applied [31]. The crystal structure was solved by direct methods and refined on *F*^2^ by full-matrix least-squares techniques with the SHELXTL-2016 program [32]. In **2**, one isopropyl group of the TAF moiety displayed conformational disorder, with its relative ratio of 0.51:0.49 refined for the disordered domains. The H atoms on the three O atoms (O8A, O6B, and O8B) of fumarate molecules and H on N4B (hydrogen-bonded to the deprotonated carboxylate of fumarate) were located from the difference Fourier map with their coordinates refined freely while their thermal parameters were constrained to *U*_iso_(H) = 1.2*U*_eq_(O). The location of the two hydrogen atoms on the coordinated water was calculated by the Calc-OH program in WinGX suite [33], their O–H distances were further restrained to O–H = 0.83 Å, and their thermal parameters were constrained to *U*_iso_(H) = 1.2*U*_eq_(O). The H atom on the –NH_2_ group was added by HFIX 93.

Crystallographic data have been deposited with the Cambridge Crystallographic Data Center as supplementary publication numbers CCDC 2040405. These data can be obtained free of charge from the Cambridge Crystallographic Data Centre via www.ccdc.cam.ac.uk/data_request/cif. A summary of the key crystallographic data is listed in Table 1.

## 4. Conclusions

To summarize, we have, for the first time, authenticated the structure of the previously elusive TAF monofumarate by single-crystal X-ray crystallography. The structural analysis shows that two pairs of TAF fumarate and one aqua molecule presented in the asymmetric unit of the crystal, where only one of the four protons from the two fumaric acids migrated to the aromatic N atom of the adenine moiety, endorsing the crystals as a mixed form featuring 75% co-crystal and 25% salt. Since the co-crystal and salt are distinctive classes of pharmaceutical solids and have significant regulatory implications, our work here exhibits a definitive advantage in clarifying the real identity of the drug when other spectroscopic tools fail. In addition, our work here may also serve as a cautionary tale for colleagues working on TAF-based products when defining the nature of their drug formulations, from both fundamental and application perspectives.

## Figures and Tables

**Table 1:** Summary of the crystallographic data for **2**.

Empirical Formula	C_50_H_68_N_12_O_19_P_2_
Temperature	296 (2) K
Formula weight	1203.10
Crystal system	monoclinic
Space group	*P*2_1_
*a*/Å	9.7367 (3)
*b*/Å	18.1659 (5)
*c*/Å	16.7114 (5)
*α*/°	90
*β*/°	98.673 (3)
*γ*/°	90
*V*/Å^3^	2922.04 (15)
*Z*	2
*D_c_*/(g cm^−3^)	1.367
*μ* (Mo-Kα)/mm^−1^	0.157
*F*(000)	1268
Total reflections	69,895
Unique reflections	11,469
No observations	9900
No parameters	803
Flack parameter	0.00 (4)
*R*_int_	0.0586
*R* ^1^	0.0430
*wR* ^2^	0.0985
*GOF* ^3^	1.040

^1^*R*_1_ = Σ||F_o_| − |F_c_||/Σ|F_o_|, ^2^ *wR*_2_ = {Σ[ω(F_o_^2^ − F_c_^2^)^2^]/Σ[ω(F_o_^2^)^2^]}^1/2^, and ^3^ *GOF* = {Σ[ω(F_o_^2^ − F_c_^2^)^2^]/(*n* − *p*)}^1/2^, where *n* is the number of reflections, and *p* the total number of parameters refined.

## Supplementary Materials

The following are available online at https://www.mdpi.com/1422-0067/21/23/9213/s1.
