# Supplementary material for: On the Single-Crystal Structure of Tenofovir Alafenamide Mono-Fumarate: A Metastable Phase Featuring a Mixture of Co-Crystal and Salt
Source: Int J Mol Sci. 2020 Dec 3;21(23):9213. doi: 10.3390/ijms21239213 (PMC7730268; doi:10.3390/ijms21239213)
Supplement: Supplementary file 1 [file ijms-21-09213-s001.pdf]

Supplementary Information for

On the Single-Crystal Structure of Tenofovir Alafenamide Mono-fumarate: A Metastable Phase Featuring A Mixture of Co-crystal and Salt

Jian–Wei Wang, Lu Liu, Ka–Xi Yu, Hong–Zhen Bai, Jun Zhou, Wen–Hua Zhang, Xiurong Hu and Guping Tang*

Table of Contents

Fig. S1 The ^{1}H nuclear magnetic resonance (NMR) of tenofovir alafenamide monofumarate hemihydrate (**2**) in DMSO-d_6 solution. 2

Fig. S2 The Fourier-transform infrared (FT−IR) spectra of tenofovir alafenamide hemi-fumarate (**1**, a), and tenofovir alafenamide monofumarate hemihydrate (**2**, b). 3

Fig. S3 The TGA−DSC curves of tenofovir alafenamide hemi-fumarate (**1**, a), and tenofovir alafenamide monofumarate hemihydrate (**2**, b). Red code for DSC curve, Blue for DSC-TGA. 4

Table S1 Selected bond distances (Å) and angles (°) for tenofovir alafenamide (TAF), tenofovir alafenamide hemi-fumarate (**1**) and tenofovir alafenamide monofumarate hemihydrate (**2**). 5

Table S2 Torsion angles (°) for tenofovir alafenamide (TAF), tenofovir alafenamide hemi-fumarate (**1**), and tenofovir alafenamide fumarate hemihydrate (**2**).......... 7

Table S3 Hydrogen−bonding tables (Å and °) for tenofovir alafenamide (TAF), tenofovir alafenamide hemi-fumarate (**1**) and tenofovir alafenamide fumarate hemihydrate (**2**). 8

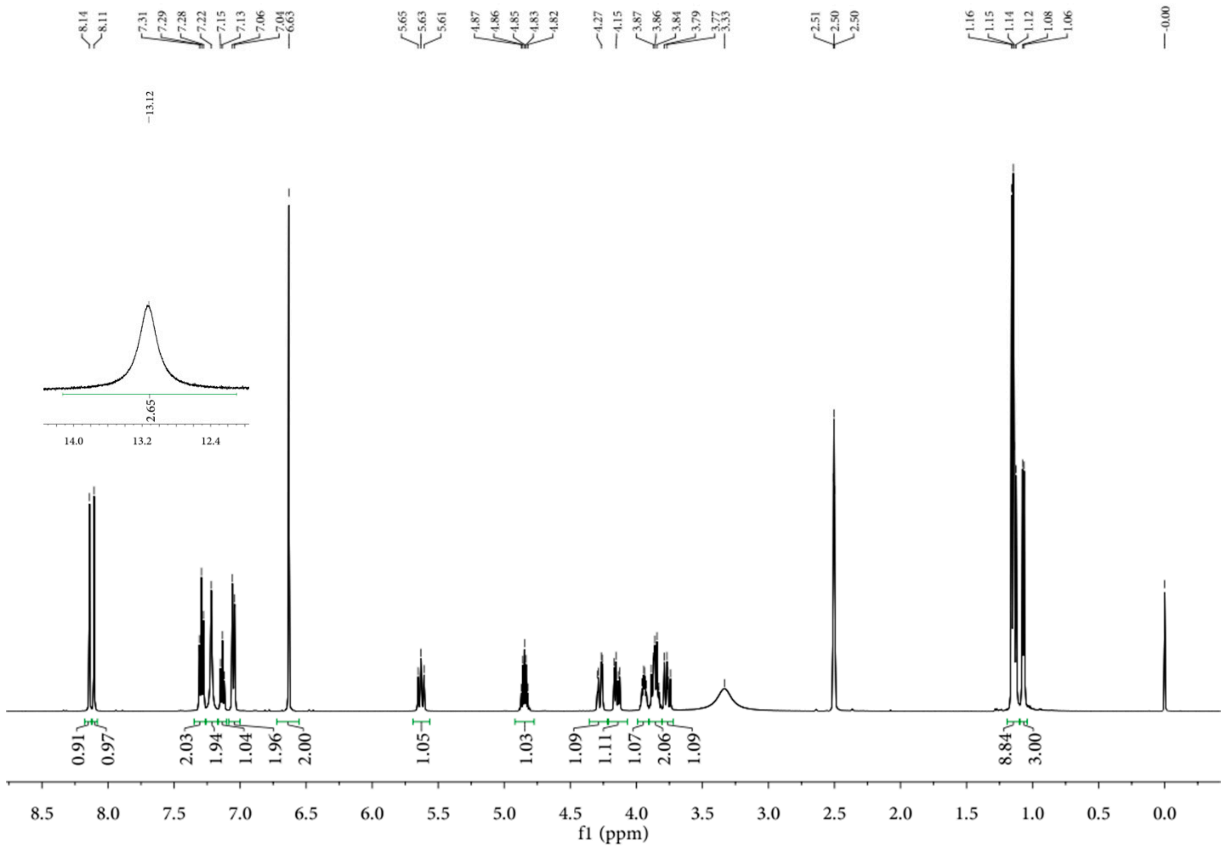

Figure S1 The ^{1}H nuclear magnetic resonance (NMR) of tenofovir alafenamide monofumarate hemihydrate (**2**) in DMSO-d_6 solution.

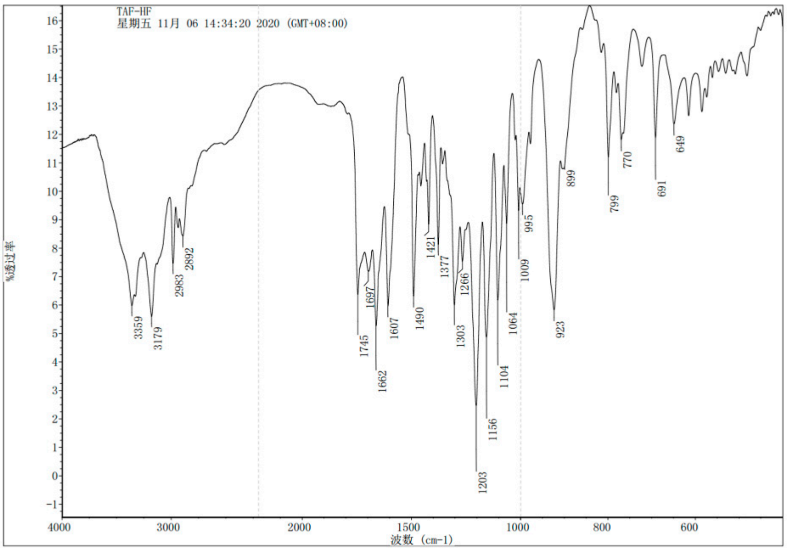

(a)

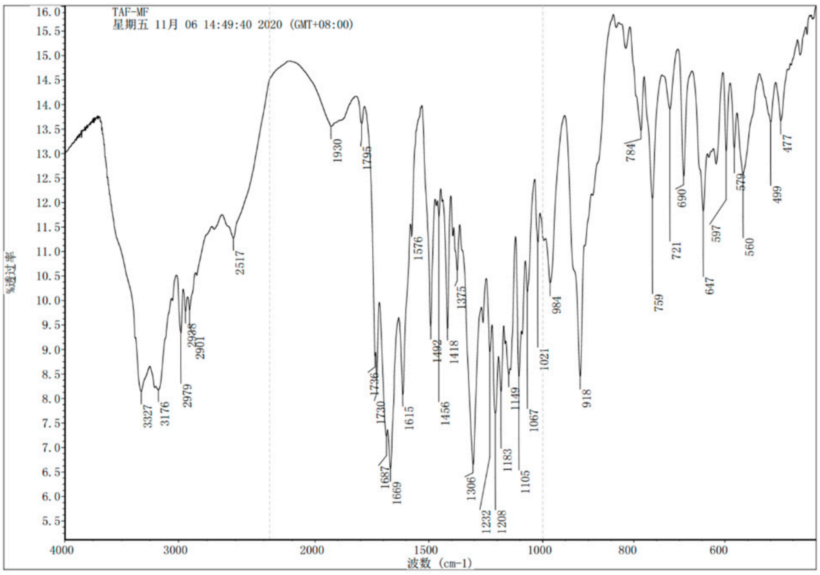

(b)

Figure S2 The Fourier-transform infrared (FT−IR) spectra of tenofovir alafenamide hemi-fumarate (**1**, a), and tenofovir alafenamide monofumarate hemihydrate (**2**, b).

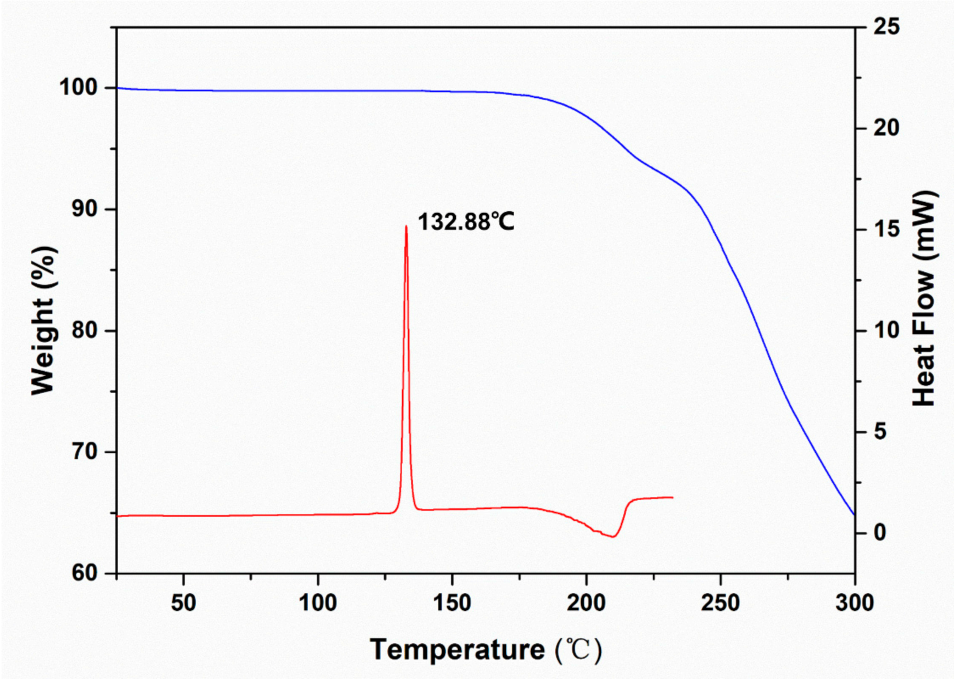

(a)

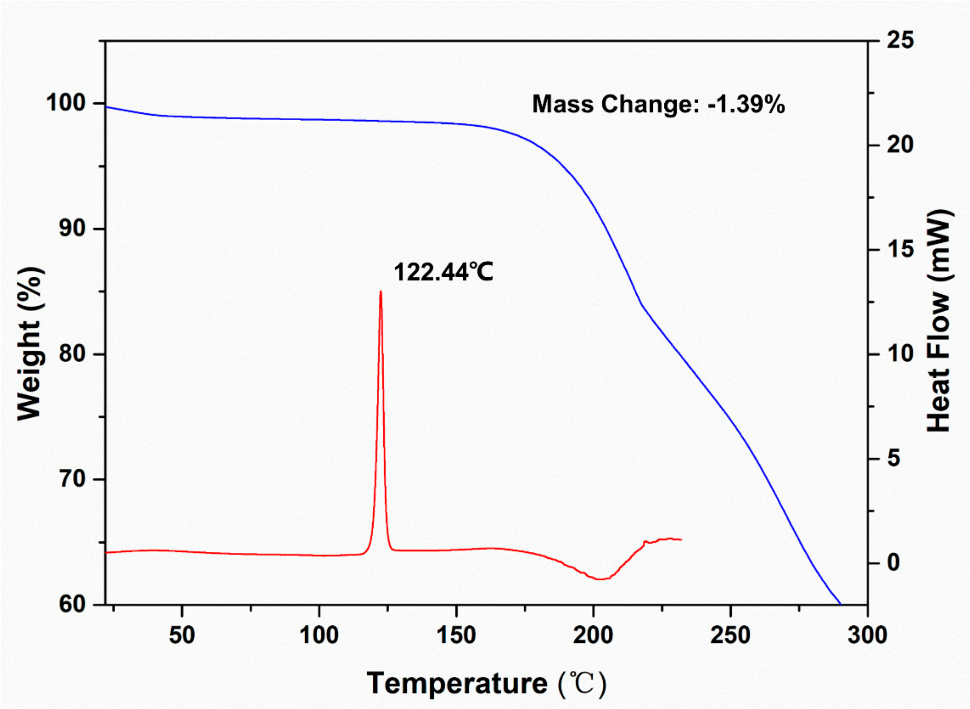

(b)

Figure S3 The TGA−DSC curves of tenofovir alafenamide hemi-fumarate (**1**, a), and tenofovir alafenamide monofumarate hemihydrate (**2**, b). Red code for DSC curve, Blue for DSC-TGA.

Table S1 Selected bond distances (Å) and angles (°) for tenofovir alafenamide (TAF), tenofovir alafenamide hemi-fumarate (**1**) and tenofovir alafenamide monofumarate hemihydrate (**2**).

**TAF**			
P(1)–O(1)	1.466(2)	P(1)–O(2)	1.596(2)
P(1)–N(10)	1.627(3)	P(1)–C(12)	1.812(3)
O(1)–P(1)–O(2)	116.42(14)	O(1)–P(1)–N(10)	112.30(14)
O(2)–P(1)–N(10)	105.68(13)	O(1)–P(1)–C(12)	110.96(15)
O(2)–P(1)–C(12)	98.50(15)	N(10)–P(1)–C(12)	112.17(15)
**tenofovir alafenamide hemi-fumarate (1)**			
P(1)–O(1)	1.473(2)	P(1)–O(2A)	1.532(4)
P(1)–N(10)	1.618(2)	P(1)–C(12)	1.799(5)
O(6)–C(25)	1.310(3)	O(7)–C(25)	1.214(4)
O(1)–P(1)–O(2A)	120.9(2)	O(1)–P(1)–N(10)	112.06(12)
O(2A)–P(1)–N(10)	108.0(2)	O(1)–P(1)–C(12)	110.46(16)
O(2A)–P(1)–C(12)	91.8(3)	N(10)–P(1)–C(12)	112.0(2)
**tenofovir alafenamide fumarate hemihydrate (2)**			
P(1A)–O(3A)	1.471(3)	P(1A)–O(4A)	1.601(3)
P(1A)–N(1A)	1.629(4)	P(1A)–C(13A)	1.803(4)
P(1B)–O(3B)	1.463(3)	P(1B)–O(4B)	1.597(3)
P(1B)–N(1B)	1.624(4)	P(1B)–C(13B)	1.814(4)
O(6A)–C(22A)	1.277(4)	O(7A)–C(22A)	1.239(4)
O(8A)–C(25A)	1.314(4)	O(9A)–C(25A)	1.215(5)
O(6B)–C(22B)	1.308(4)	O(7B)–C(22B)	1.227(5)
O(8B)–C(25B)	1.313(4)	O(9B)–C(25B)	1.223(5)
N(4B)–H(4N)	0.83(4)	O(8A)–H(8OA)	0.83(5)
O(6B)–H(6OB)	1.02(5)	O(8B)–H(8OB)	0.87(5)
O(3A)–P(1A)–O(4A)	115.44(17)	O(3A)–P(1A)–N(1A)	113.42(18)
O(4A)–P(1A)–N(1A)	104.80(16)	O(3A)–P(1A)–C(13A)	110.83(19)
O(4A)–P(1A)–C(13A)	103.70(18)	N(1A)–P(1A)–C(13A)	107.90(19)
O(3B)–P(1B)–O(4B)	116.3(2)	O(3B)–P(1B)–N(1B)	114.77(19)

O(4B)−P(1B)−N(1B)	101.91(18)	O(3B)−P(1B)−C(13B)	113.8(2)
O(4B)−P(1B)−C(13B)	104.4(2)	N(1B)−P(1B)−C(13B)	104.1(2)

Table S2 Torsion angles (°) for tenofovir alafenamide (TAF), tenofovir alafenamide hemi-fumarate (**1**), and tenofovir alafenamide fumarate hemihydrate (**2**).

TAF			
P(1)–N(10)–C(13)–C(15)	−90.3(3)	C(10)–O(3)–C(12)–P(1)	−123.0(3)
P(1)–O(2)–C(19)–C(20)	−69.9(4)	P(1)–O(2)–C(19)–C(24)	110.6(4)
C(12)–O(3)–C(10)–C(9)	170.3(2)	N(9)–C(9)–C(10)–O(3)	−66.1(3)
tenofovir alafenamide hemi-fumarate (**1**)			
P(1)–N(10)–C(13)–C(15)	−98.6(3)	P(1)–O(2A)–C(19A)–C(20A)	−93.5(6)
P(1)–O(2A)–C(19A)–C(24A)	90.1(7)	C(12)–O(3)–C(10)–C(9)	172.8(3)
C(10)–O(3)–C(12)–P(1)	−162.9(3)	N(9)–C(9)–C(10)–O(3)	−55.8(3)
tenofovir alafenamide fumarate hemihydrate (**2**)			
P(1A)–N(1A)–C(1A)–C(2A)	−90.1(4)	P(1B)–N(1B)–C(1B)–C(2B)	−97.2(4)
P(1A)–O(4A)–C(7A)–C(8A)	−148.7(3)	P(1B)–O(4B)–C(7B)–C(8B)	−102.0(5)
P(1A)–O(4A)–C(7A)–C(12A)	33.1(5)	P(1B)–O(4B)–C(7B)–C(12B)	83.1(5)
C(14A)–O(5A)–C(13A)–P(1A)	−149.9(3)	C(14B)–O(5B)–C(13B)–P(1B)	174.0(3)
C(13A)–O(5A)–C(14A)–C(15A)	174.8(3)	C(13B)–O(5B)–C(14B)–C(15B)	154.5(3)
O(5A)–C(14A)–C(15A)–N(2A)	−62.1(4)	O(5B)–C(14B)–C(15B)–N(2B)	59.4(4)

Table S3 Hydrogen–bonding tables (Å and °) for tenofovir alafenamide (TAF), tenofovir alafenamide hemi-fumarate (**1**) and tenofovir alafenamide fumarate hemihydrate (**2**).

D–H···A	d(D–H)	d(H···A)	d(D···A)	∠(DHA)
**TAF**				
N(6)–H(6A)...N(1)#1	0.859(13)	2.165(18)	3.005(4)	166(4)
N(6)–H(6B)...N(7)#2	0.857(13)	2.275(14)	3.132(4)	178(4)
N(10)–H(10)...O(1)#3	0.860(13)	2.24(2)	3.040(3)	155(3)
Symmetry codes: #1 *x* + 1/2, −*y* + 1/2, −*z* + 1; #2 *x* − 1/2, −*y* + 1/2, −*z* + 1; #3 *x* + 1/2, −*y* + 3/2, −*z* + 1.				
**tenofovir alafenamide hemi-fumarate (1)**				
N(6)–H(6A)...O(1)#1	0.857(13)	2.066(16)	2.907(3)	167(3)
N(6)–H(6B)...O(7)	0.867(13)	2.107(14)	2.973(3)	176(3)
N(10)–H(10)...N(1)#2	0.864(13)	2.085(14)	2.949(3)	178(3)
O(6)–H(6)...N(7)	1.0274(19)	1.571(2)	2.576(3)	164.73(14)
Symmetry codes: #1 −*y* + 3/2, *x* + 1/2, *z* + 1/2; #2 *y* − 1/2, −*x* + 3/2, *z* − 1/2.				
**tenofovir alafenamide fumarate hemihydrate (2)**				
N(4B)–H(4N)...O(6A)#1	0.83(4)	1.81(5)	2.639(4)	174(4)
O(6B)–H(6OB)...N(4A)#2	1.02(5)	1.57(5)	2.576(4)	167(4)
O(8A)–H(8OA)...N(3A)	0.83(5)	1.88(5)	2.700(4)	168(5)
O(8B)–H(8OB)...N(3B)	0.87(5)	1.79(5)	2.654(4)	172(4)
O(1W)–H(1W)...O(3B)	0.83	2.55	3.003(7)	115.7
O(1W)–H(2W)...N(5A)#3	0.83	2.20	2.987(7)	159.1
N(1A)–H(1AA)...O(6A)#2	0.86	2.25	3.044(4)	153.1
N(1B)–H(1BA)...O(3A)#4	0.86	2.16	2.990(5)	163.2
N(6A)–H(6AD)...O(7B)#3	0.86	1.95	2.801(4)	169.6
N(6A)–H(6AE)...O(9A)	0.86	1.94	2.785(4)	165.6
N(6B)–H(6BD)...O(7A)#1	0.86	1.88	2.732(4)	173.1
N(6B)–H(6BE)...O(9B)	0.86	1.95	2.785(4)	164.5
Symmetry codes: #1 *x* + 1, *y* + 1, *z*; #2 *x* + 1, *y*, *z*; #3 *x* − 1, *y*, *z*; #4 −*x*, *y* + 1/2, −*z* + 1.				
